# Thermal Performance Evaluation of Encapsulated Phase Change Materials Exposed to Contact Heat and Radiant Heat

**DOI:** 10.3390/ma18184271

**Published:** 2025-09-12

**Authors:** Adam K. Puszkarz, Emilia Śmiechowicz, Waldemar Machnowski

**Affiliations:** 1 Textile Institute, Lodz University of Technology, 116 Zeromskiego Street, 90-924 Lodz, Poland; waldemar.machnowski@p.lodz.pl; 2 Department of Mechanical Engineering, Informatics and Chemistry of Polymer Materials, Lodz University of Technology, 116 Zeromskiego Street, 90-924 Lodz, Poland; emilia.smiechowicz@p.lodz.pl

**Keywords:** phase change materials, n-octadecane, n-hexadecane, DSC, thermal cycling stability, contact heat, radiant heat, micro-CT

## Abstract

This article describes research on two encapsulated phase change materials (PCMs) from the alkane group (n-hexadecane and n-octadecane) with phase transition temperatures of 18.2 °C and 28.2 °C, respectively. The main goal of the study was to determine the internal structure and basic thermal properties of both types of macrocapsules in terms of their potential applications. The internal structure of the macrocapsules was characterized using non-destructive statistical quantitative analysis performed using X-ray microtomography (micro-CT). Differential scanning calorimetry (DSC) was used to determine the phase transition temperatures, thermal cycling stability, and phase transition enthalpies of both PCMs. The macrocapsules were tested in two experiments, simulating the conditions of their potential application by exposing them to contact heat and radiant heat. Structural analysis showed that the macrocapsules differ significantly in PCM content (77% n-hexadecane and 88% n-octadecane) and porosity (19% and 10%, respectively). According to the DSC results, the macrocapsules with n-octadecane exhibited a significantly wider phase transition range and a greater ability to store latent heat indicated by its higher enthalpy by about 30 J·g^−1^ than those with n-hexadecane. The results of experiments involving PCM exposure to contact heat and radiant heat demonstrated the potential applications of the macrocapsules in thermal packaging, building, and protective clothing.

## 1. Introduction

Interest in phase change materials (PCMs) has been continuing for over 30 years. This interest is manifested by a still growing number of publications on both manufacturing, testing properties, and dissemination of the use of PCM materials [1,2,3]. In recent years, the number of publications on PCMs has reached over 10,000 articles per year [4]. Materials called PCMs are a group of inorganic, organic and eutectic substances, which undergo a phase change when heating or cooling them at temperatures close to the phase transition temperature. The processes of changing the state of the substances, like melting or condensation, occur as a result of the heat absorption or heat release, which are known as latent heat or transition heat [5,6,7].

From a practical point of view, for the effective use of PCM materials, the most important fact is that the phase transition processes of these materials occur at a near constant temperature. It is this temperature stability of the system during heat supply to it (or heat extraction from it) that is the essence of the unique thermal properties of PCMs, which create many possibilities for their practical applications.

When the PCM is in a solid state (below its melting temperature), its temperature increases as it absorbs heat. If the PCM continues to be heated and reaches the phase transition temperature, it will start to absorb heat without changing its temperature. The observed phenomenon of temperature constancy of the PCM material during heat absorption results from the fact that all the heat supplied to the PCM material is converted into a change in the material’s state (from solid to liquid). The temperature of the PCM, when heat is still supplied to it, begins to rise again only when the entire material has converted into a liquid (completely melted).

The above-described behavior of PCMs indicates that introducing these materials into the structure of various systems/objects can effectively improve their thermal properties, such as thermal insulation in both hot and cold environments, e.g., can slow down their heating when they are used in ambient conditions with excessively high temperatures. An example of this is introducing PCMs to create textile materials intended for firefighter clothing with enhanced thermal protective properties [8,9]. So-called smart textiles (with thermoregulation features) were among the first PCM applications. Fabrics containing PCM microcapsules and multilayer textile assemblies with inserted PCM macrocapsules are used to make a wide variety of special functional garments, including space suits, subjected to large temperature fluctuations [3,10]. The term “PCM capsule” covers a wide range of products in which the phase change material (core) is contained within a shell (acts as a wall) that prevents the mass exchange but allows the heat exchange with the environment. Enclosing the phase change material within a shell allows it to be incorporated into various systems/elements, as it prevents direct contact with the environment and PCM leakage when the solid phase is melted. The specific application possibilities of encapsulated PCMs depend not only on the thermal parameters of the phase change material, but also on the internal structure of the capsule and material of its shell [3]. Based on the diameter size, the PCM capsules are divided into three types: nano (<1 μm), micro (>1 μm), macro (>1000 μm) [4].

PCMs are also incorporated into various building materials such as bricks, gypsum boards, and concrete in order to increase energy efficiency of buildings and stabilize internal temperature in the rooms. Due to the presence of PCMs, the aforementioned building elements, in the summer months, absorb excess heat during the day and release it at night, improving thermal comfort in the rooms and mitigating cooling/heating energy demand [3,4,5].

Many organic PCMs are very useful in buildings applications, as well as in electric vehicles and textile applications, but they have a major drawback, namely they are flammable [11,12]. In all the above-mentioned applications, flame-resistance is, of course, the critical requirement. Therefore, materials containing organic PCMs (paraffins, fatty acids, esters, alcohols) intended for use in construction, automotive, and protective clothing industries must pass flammability certifications to qualify as safe, high-quality components. Many research studies in recent years have been devoted to the methods for achieving flame retardancy for organic PCMs. As a result, methods for integrating various flame retardants with bulk and encapsulated PCMs have been developed that enable the production of organic PCMs with flame-retardancy properties [11,13,14].

PCMs can be very useful for storing and transporting temperature-sensitive medical items (pharmaceuticals, blood products, vaccinations) [15,16,17] and foods [18,19,20], protecting them from both excessive heat and cooling.

A relatively new and very important area of application for PCMs is their use in renewable energy systems. Thanks to their high latent heat, PCMs may be a lower-cost solution that increases the general effectiveness of solar equipment and systems. They are appropriate for a wide range of applications, from small-scale grid systems to household energy storage [3,21,22]. A periodic excess of electricity produced from renewable sources, during sunny and windy days, can be used for PCM heating to cause its phase transition (from solid to liquid). In this way, the energy is stored in the PCM substance. This PCM material now becomes a reliable source of energy that can be used when its production is not very effective, e.g., at night and in the absence of wind [4,23]. The examples of PCM applications presented indicate that phase change materials can contribute to reducing the consumption of fossil fuels, and thus reduce greenhouse gas emissions.

A review of publications clearly shows that PCMs can be used effectively in a very wide range of applications. However, it should be noted that PCMs are not commonly used in real-world applications at present; their use is still significantly lower than the enormous application potential of these materials. Therefore, it is reasonable to undertake any research whose results will improve our understanding of how PCM materials behave under specific thermal conditions, when heat is transferred to them by different mechanisms.

In the current article, the authors selected macrocapsules containing phase change materials (n-hexadecane and n-octadecane) with a polyurethane (PU) shell as the subject of their study. In order to more easily observe the differences between the tested PCM capsules, they were tested as such and not as capsules introduced into some system/object. The aim of the study was to investigate the internal structure of PCM macrocapsules (using X-ray microtomography) and the basic thermal parameters of these macrocapsules (using DSC), as well as to examine the kinetics of phase transitions of both PCM macrocapsules in two experiments, simulating the conditions of their potential application by subjecting them to contact heat and radiant heat.

## 2. Materials and Methods

### 2.1. Materials

**Figure 1:**
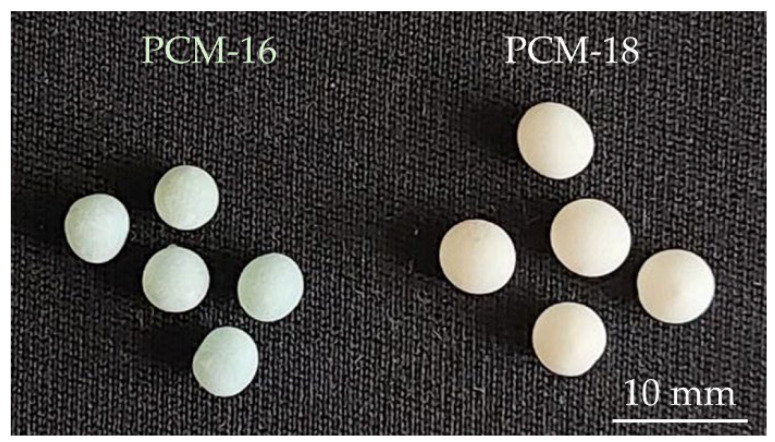
Photos of tested macrocapsules.

The study examined PCM-16 and PCM-18 macrocapsules (Microtek Laboratories, Moraine, OH, USA) containing two different phase change materials (PCMs): n-hexadecane (C_16_H_34_) and n-octadecane (C_18_H_38_), respectively. Due to their high latent heat, low cost, chemical stability, and non-toxicity, paraffins are one of the most commonly used types of PCM materials [4,10]. The PCM material in both tested macrocapsules was contained within multiple microcapsules. Both the macrocapsule shells and microcapsule shells are made of polyurethane. The PCM-16 and PCM-18 macrocapsules (shown in Figure 1) had a rounded, spherical shape with diameters not exceeding 4 mm and 5 mm, respectively.

The basic physical properties of PCM-16 and PCM-18 macrocapsules are presented in Table 1.

### 2.2. Methods

#### 2.2.1. Analysis of the Internal Structure of Macrocapsules

To determine the structure of the macrocapsules, the authors used high-resolution X-ray microtomography (micro-CT, Bruker, Kontich, Belgium), which they successfully applied in their studies of textiles and fiber composites used in protective clothing [28,29,30]. This measurement technique is not commonly used in the study of capsulated phase change materials, although it allows for obtaining a lot of valuable information about the structural parameters of these capsules that may have a key impact on their functional thermal properties. Morphology characteristics of the microstructure macrocapsules of both types, scanned under conditions: X-ray source voltage 50 kV, X-ray source current 200 µA, and voxel size 4 µm. A rotation step of 0.2° without a filter was applied. Due to the different absorption of X-rays by alkanes, polyurethane, and air, it was possible to identify and quantitatively analyze the components of the macrocapsules. Statistical distributions of microcapsules’ diameter within the macrocapsules, macrocapsule shell thicknesses, and macrocapsule pore size distributions were determined. Furthermore, the volumetric composition of the macrocapsules, including the air filling the closed micropores, was calculated.

#### 2.2.2. Analysis of Thermal Properties of Macrocapsules

The phase transition enthalpies, phase change temperatures, and cycling stability of the PCMs macrocapsules were studied with a DSC 6 calorimeter (Perkin-Elmer, Waltham, MA, USA) in the range of 0 °C to 50 °C with a heating/cooling rate of 5 °C min^−1^. The mass of the samples was 12 mg. The computer-controlled DSC 6 calorimeter allows for heat flux measurements of energetic effects occurring in the sample.

#### 2.2.3. Exposure of Macrocapsules to Contact Heat

**Figure 2:**
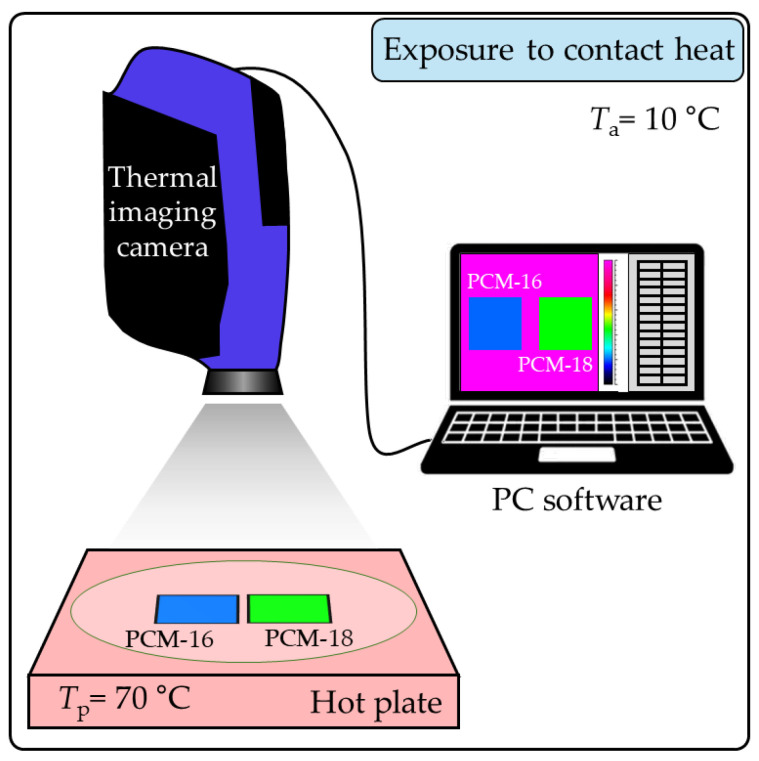
Scheme of PCM packets’ exposure to contact heat experiment [8].

To investigate the kinetic phase transitions of PCM-16 and PCM-18 macrocapsules exposed to contact heat, an experiment (scheme in Figure 2) was conducted using a heated flat plate (e-G51HP07C Guardian 5000 model made by OHAUS Europe GmbH, Nänikon, Switzerland) and a thermal imaging camera (FLIR SC 5000 model made in Wilsonville, OR, USA). Macrocapsules of both PCM types formed two separate single-layer packets with a square surface area of 3 cm × 3 cm. The packets lay flat on the hot plate surface, and each macrocapsule was in direct contact with the hot plate with a constant temperature of 70 °C.

Due to the 60 degree temperature difference between the plate (70 °C) and the surroundings (10 °C), a heat flow was generated from the hot plate toward the cooler environment through the macrocapsules. As a result, the macrocapsules gradually increased their temperature until they reached a maximum constant temperature. The heating process of the macrocapsules was recorded in real time by the thermal imaging camera placed above the plate.

#### 2.2.4. Exposure of Macrocapsules to Radiant Heat

**Figure 3:**
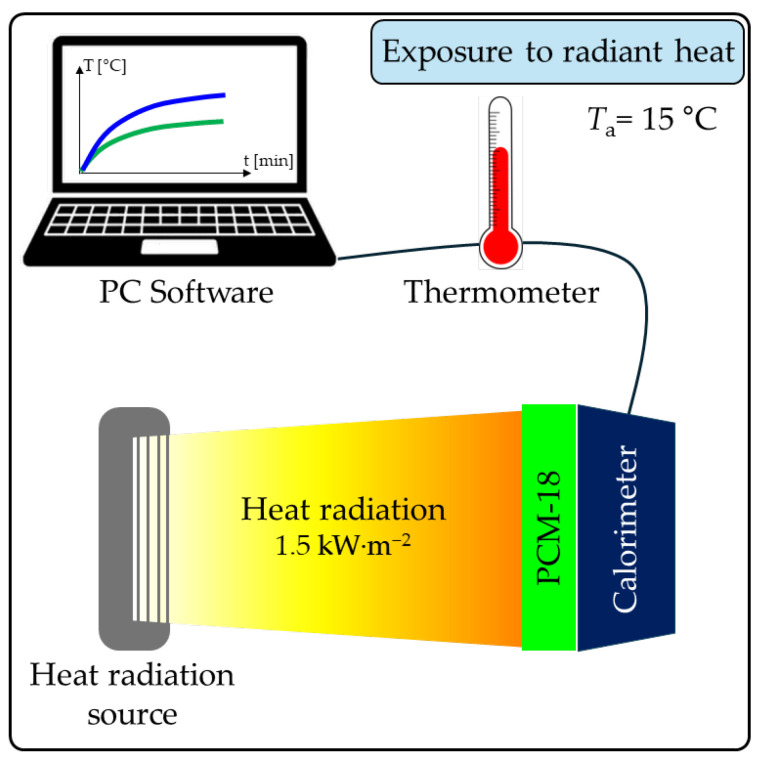
Scheme of PCM packets’ exposure to radiant heat experiment [8].

To evaluate the kinetic phase transitions of PCM-16 and PCM-18 macrocapsules exposed to thermal radiation, an experiment was conducted (scheme in Figure 3). In this method, macrocapsules of both PCM types formed two separate single-layer packets. These packages, with a dimension of approximately 5 cm × 5 cm, were oriented vertically, with one side pressed against a flat calorimeter sensor, and the other side exposed to a radiant heat flux density of 1.5 kW·m^−2^ emitted by a heat radiation source. This intensity of heat radiation is similar to the total amount of solar electromagnetic radiation, reaching the Earth’s upper atmosphere called Total Solar Energy (TSI), which is 1.362 kW·m^−2^ [31]. Therefore, such heat radiation may potentially affect the protective clothing intended for some workers employed in agriculture and road construction; this level of heat radiation may also fall on some elements of building facades. And it is precisely this protective clothing and the mentioned building elements that are potential objects for the introduction of tested PCM macrocapsules.

As a result of the incident radiant heat flux, the macrocapsule packages gradually heated up, and their temperature was measured in real time by a thermometer connected to the calorimeter. The experiment was conducted at an ambient temperature of 15 °C.

## 3. Results and Discussion

### 3.1. Analysis of the Internal Structure of Macrocapsules

**Figure 4:**
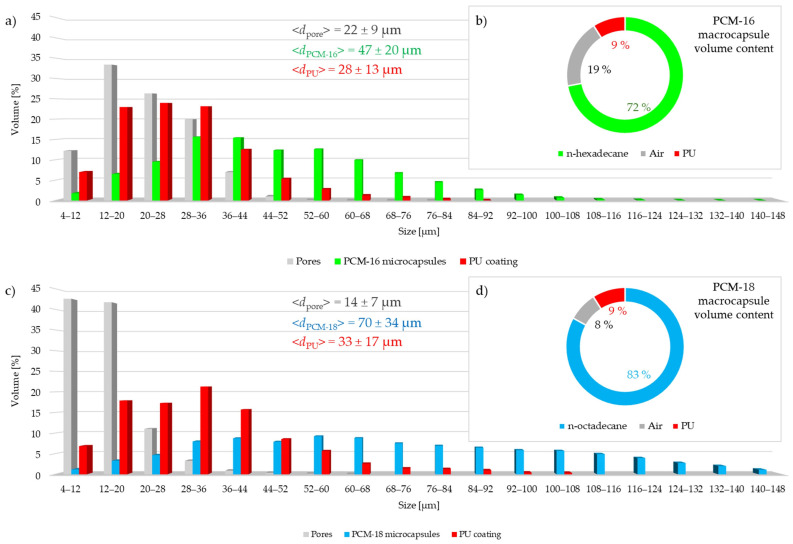
Results of micro-CT structural analysis of tested PCM macrocapsules: (**a**) Distributions of microcapsules diameter, shell thickness, and pore sizes enclosed inside PCM-16 macrocapsules; (**b**) volumetric composition of the PCM-16 macrocapsule; (**c**) Distributions of microcapsules diameter, shell thickness, and pore sizes enclosed inside PCM-18 macrocapsules; (**d**) volumetric content of the PCM-18 macrocapsule.

Figure 4 presents the results of the internal structure analysis of PCM-16 and PCM-18 macrocapsules obtained by X-ray microtomography.

Based on the obtained results, presented in Figure 4a,c, it can be observed that PCM-16 macrocapsules are composed of significantly smaller microcapsules than PCM-18 macrocapsules. The diameter of PCM-16 microcapsules *d*_PCM-16_ ranges from 4 µm to 148 µm (average value <*d*_PCM-16_> is 47 µm), while the diameter of PCM-18 microcapsules *d*_PCM-18_ varies within the same range, but the average value <*d*_PCM-18_> is 70 µm. Similar distributions of polyurethane shell thickness *d*_PU_ were observed for both types of macrocapsules, with the average value for PCM-16 macrocapsules <*d*_PU_> being 28 µm and for PCM-18 macrocapsules being 33 µm. A significant difference in pore size *d*_pore_ was observed between the two macrocapsules tested. In the case of PCM-16 macrocapsules, the pore size ranges from 4 µm to 84 µm (average value <*d*_pore_> is 22 µm), while in PCM-18 macrocapsules the pore size ranges from 4 µm to 68 µm, with an average value <*d*_pore_> of 14 µm. An even greater difference between the tested macrocapsules was observed in their porosity (Figure 4b,d). PCM-16 macrocapsules are more than twice as porous as PCM-18 macrocapsules. Air fills 19% of the entire volume of PCM-16 macrocapsules, while in PCM-18 macrocapsules it occupies 8%. Because polyurethane occupies the same volume in both capsules (9%), these capsules differ significantly in the content of phase change materials. In the case of the PCM-16 macrocapsule, n-hexadecane occupies 72%, while in the PCM-18 macrocapsule, there is 11% more n-hexadecane (83%).

### 3.2. Analysis of Thermal Properties of Macrocapsules

**Figure 5:**
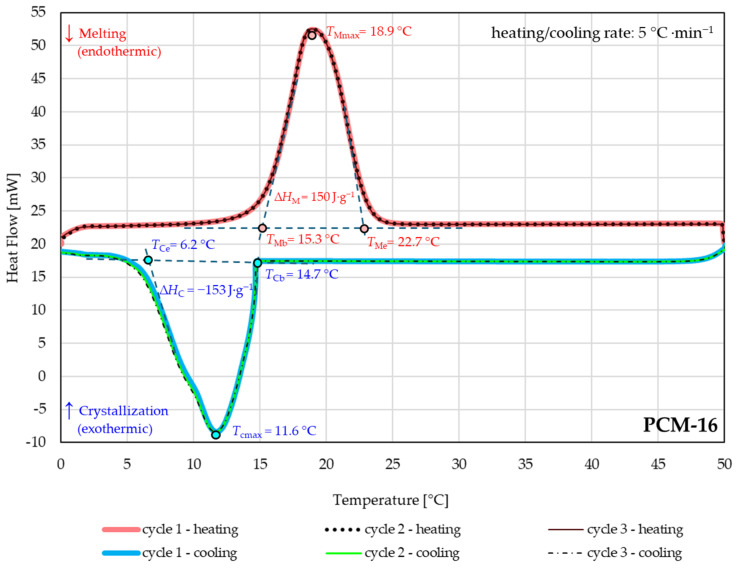
The melting and crystallization curves of the PCM-16 macrocapsules measured through the thermal reliability test.

**Figure 6:**
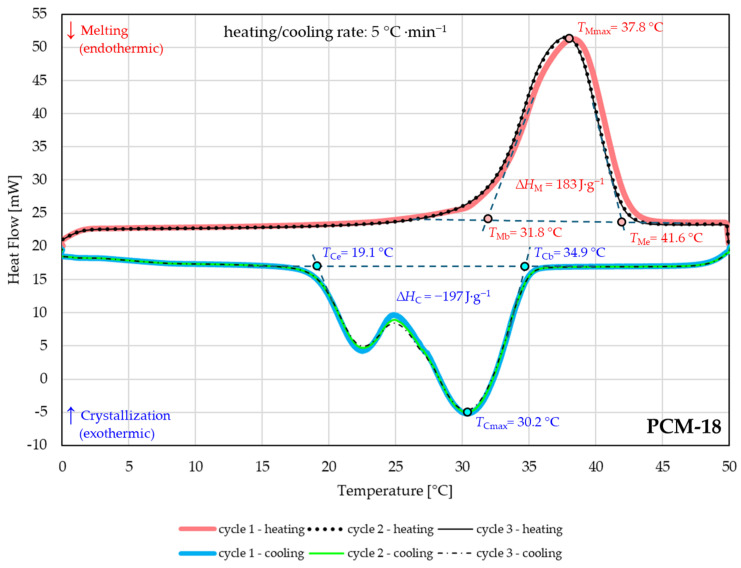
The melting and crystallization curves of the PCM-18 macrocapsules measured through the thermal reliability test.

The phase change temperatures, phase transition enthalpies, and cycling stability of PCMs macrocapsules were studied with DSC. The endothermic melting and exothermic crystallization processes of PCM macrocapsules PCM-16 and PCM-18 were estimated. Figure 5 and Figure 6 show the melting and crystallization curves with temperature ranges (melting temperature beginning: *T*_Mb_, melting temperature end: *T*_Me_ and crystallization temperature beginning: *T*_Cb_, crystallization temperature end: *T*_Ce_), melting and crystallization points (maximum temperature of melting: *T*_Mmax_, maximum temperature of crystallization: *T*_Cmax_), and enthalpies (melting enthalpy: ∆*H*_M_, crystallization enthalpy: ∆*H*_C_) of PCM-16 and PCM-18 macrocapsules. Clearly visible differences in the transition period of the tested PCMs can be noticed in Figure 5 and Figure 6.

The curve for the PCM-16 macrocapsule (n-hexadecane) showed different ranges of phase change temperatures than PCM-18 (n-octadecane). The melting process took place in the temperature range of 15.3 °C to 22.7 °C with a melting point *T*_Mmax_ = 18.9 °C and a melting enthalpy ∆*H*_M_ = 150 J·g^−1^. The crystallization temperature range was 14.7 °C to 6.2 °C with a crystallization point *T*_Cmax_ = 11.6 °C and crystallization enthalpy ∆*H*_C_ = −153 J·g^−1^ (Figure 5). The PCM-18 macrocapsule had a single melting peak and the melting process occurred in the temperature ranges of 31.8 °C to 41.6 °C with a melting point *T*_Mmax_ = 37.8 °C and melting enthalpy ∆*H*_M_ = 183 J·g^−1^ (Figure 6). The exothermic process took place in the temperature range of 34.9 °C to 19.1 °C with a crystallization point *T*_Cmax_ = 30.2 °C and crystallization enthalpy ∆*H*_C_ = −187 J g^−1^.

In the case of the PCM-18 macrocapsule with n-octadecane, a greater capacity for storing latent heat was evident; the enthalpy of melting and enthalpy of crystallization were higher by more than 30 J g^−1^ than those of PCM-16.

The thermal reliability of both PCMs macrocapsules for three heating and cooling cycles for the temperature range of 0 °C to 50 °C was performed. The only slight shift was observed in the first heating cycle during heat absorption of PCM-18. The deviation in the curve may be related to the change in the conformation and distribution of n-octadecane inside the capsule during its first melting and crystallization, which is also observed by other research [32]. It is assumed that only during the second heating cycle does the melting temperature stabilize; the heating and cooling curves for the second and third cycles overlap and behave in the same way. In the case of the graph for PCM-16 (Figure 5), repeatability of the cycles was observed.

The thermal reliability test showed that significant changes in the peak temperatures of the melting and crystallization processes (*T*_Mmax_, *T*_Cmax_) and the enthalpies of the phase transitions were not observed.

The significantly different ranges of phase transition temperatures of the tested PCM macrocapsules (Figure 5 and Figure 6 and Table 2) allow us to direct the PCMs to potentially different areas of applications. The heat absorption and heat release ranges of PCM-16 are 15.3 °C to 22.7 °C and 14.7 °C to 6.2 °C, respectively. It can be observed that the melting and crystallization process of PCM-16 occurs at a much lower temperature range compared to PCM-18, which directs the tested PCMs to different application areas. For example, many medical products must be stored under specific temperature conditions, often at controlled room temperature (CRT) or normal storage conditions, defined as a Controlled Ambient Temperature (CAT) of 15 °C to 25 °C [33,34,35]. PCM-16 has the potential to be used to modify packaging to maintain room temperature within the package.

In the case of PCM-18, the process of thermal energy absorption, i.e., the melting process, occurs in the temperature range of 31.8 °C to 41.6 °C, and the thermal energy emission, the crystallization process, in the range of 34.9 °C to 19.1 °C. The noticed range of phase change temperatures of PCM-18 allows for directing the tested PCM to the areas of thermoregulatory clothing (thermoregulatory and enthalphy fibers), i.e., textile materials with functions that absorb or release heat depending on the change in environmental parameters and the level of physical activity of the user. The visible temperature range is within the temperature range covering the user’s physical activity, especially in relation to groups of athletes, whose body temperature reactions to physical activity are manifested by an increase in temperature up to a maximum of 41.5 °C [36]. The minimum physiological comfort is provided to the user by clothing that allows the skin temperature to be maintained in the normal range of 32 °C to 34 °C, without liquid sweat [37]. PCM-18 can fulfill the main function of PCMs in clothing materials, which is to minimize the heat flow between the human body and the external environment and thus maintain the skin temperature at an almost constant level considered comfortable.

### 3.3. Exposure of Macrocapsules to Contact Heat

**Figure 7:**
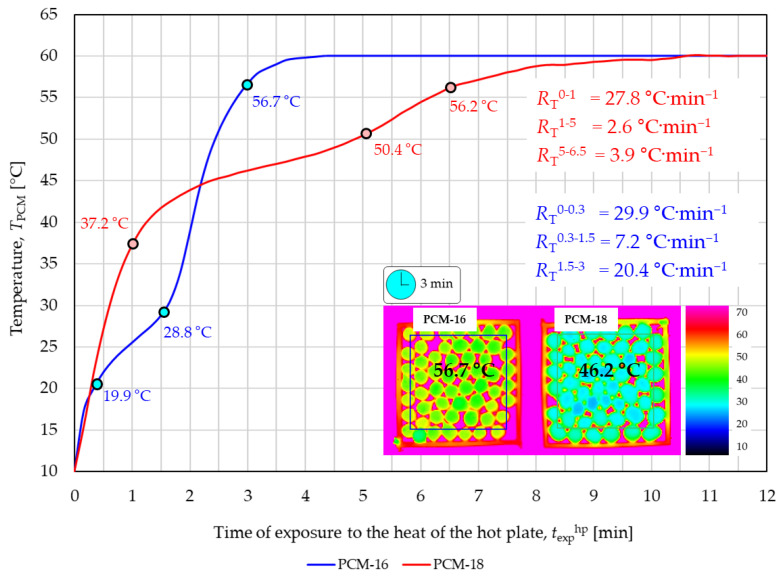
Results of PCM packets’ exposure to contact heat.

The two curves in the graph shown in Figure 7 illustrate the heating process of PCM-16 and PCM-18 macrocapsule packages exposed to the contact heat of a hot plate recorded using thermal imaging.

The temperature of both packages increased from an ambient temperature of *T*_a_ = 10 °C to reach a maximum temperature of 60 °C after approximately 10.5 min. Based on the shape of both curves, three states of phase change materials (solid, mixed, liquid) can be distinguished in both types of macrocapsules. The PCM-16 macrocapsule package heated from an initial temperature of 10 °C and after approximately 0.3 min reached a temperature of 19.9 °C, at which the melting process of n-hexadecane was initialized. At this temperature, the temperature increase rate *R*_T_ of the PCM-16 macrocapsule package decreased from *R*_T_^0−0.3^ = 29.9 °C·min^−1^ to *R*_T_^0.3−1.5^ = 7.2 °C·min^−1^. The n-hexadecane melting process continues for approximately 1.5 min, when the PCM-16 package reaches a temperature of 28.8 °C. After this time, the n-hexadecane is completely melted, and the heating rate of the PCM-16 package increases to *R*_T_^1.5−3^ = 20.4 °C·min^−1^. The PCM-18 macrocapsule package heated from an initial temperature of 10 °C and after approximately 1 min reached a temperature of 37.2 °C, at which the beginning of the melting process of n-octadecane was noticed. At this temperature, the temperature increase rate *R*_T_ of the PCM-18 macrocapsule package decreased from *R*_T_^0−1^ = 27.8 °C·min^−1^ to *R*_T_^1−5^ = 2.6 °C·min^−1^. The n-octadecane melting process continues for approximately 5 min, when the PCM-18 package reaches a temperature of 50.4 °C. After this time, the n-octadecane is completely melted, and the heating rate of the PCM-18 package increases to *R*_T_^5−6.5^ = 3.9 °C·min^−1^. The observed large difference in the duration of the mixed state of phase change material between the two types of macrocapsules is noteworthy. The melting time of n-hexadecane in PCM-16 macrocapsules was approximately 1.2 min, while the melting time of n-octadecane in PCM-18 macrocapsules was 4 min. The significant difference in the duration of the PCM melting processes in both types of macrocapsules could be caused by the significantly lower content of n-hexadecane in the PCM-16 capsule (72%) compared to the content of n-octadecane in the PCM-18 capsule (83%), as well as a significant difference in melting temperature (10 °C) between alkanes contained in the tested macrocapsules. The lower right corner of the graph in Figure 7 shows thermograms illustrating the temperature distribution of the top surface of both the PCM-16 package and PCM-18 package, recorded in the third minute of the experiment. The recorded top surface temperature of packages was the average temperature measured within a square-shaped area (with a side of approximately 2.5 cm) located in the center of the package. The recorded average temperature of each package was slightly overestimated because it also resulted from the measured temperature of the hot plate, which was visible to a thermal imaging camera between the macrocapsules.

### 3.4. Exposure of Macrocapsules to Radiant Heat

**Figure 8:**
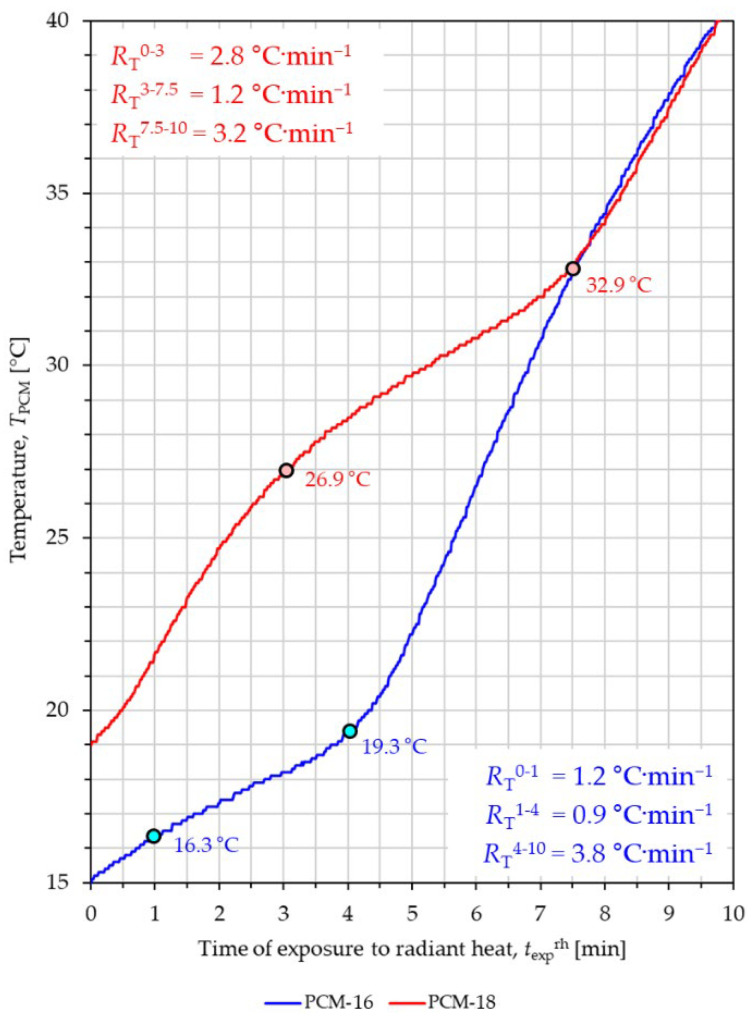
Results of PCM packets’ exposure to radiant heat.

The two curves in the graph shown in Figure 8 illustrate the heating process of PCM-16 and PCM-18 microcapsule packages exposed to the radiant heat.

The experiment’s initial temperature of both packages was selected so that it was significantly lower than the melting point of the given PCM. In the case of the package with PCM-16 macrocapsules, it was 15 °C, while in the case of the package with PCM-18 macrocapsules, it was 19 °C. As a result of incident thermal radiation, both packages reached the same temperature of 32.9 °C after 7.5 min. Based on the shape of both curves, similarly to the experiment with exposure of macrocapsules to contact heat, three states of PCMs can be distinguished in both types of macrocapsules (solid, mixed, liquid).

The PCM-16 macrocapsule package heated from an initial temperature of 15 °C and after 1 min reached a temperature of 16.3 °C, at which the melting process of n-hexadecane was observed. At this temperature, the temperature increase rate *R*_T_ of the PCM-16 macrocapsule package decreased from *R*_T_^0−1^ = 1.2 °C·min^−1^ to *R*_T_^1−4^ = 0.9 °C·min^−1^. The n-hexadecane melting process continues for approximately 4 min, when the PCM-16 package reaches a temperature of 19.3 °C. After this time, the n-hexadecane is completely melted, and the heating rate of the PCM-16 package increases to *R*_T_^4−10^ = 3.8 °C·min^−1^.

The PCM-18 macrocapsule package heated from an initial temperature of 19 °C and after approximately 3 min reached a temperature of 26.9 °C, at which the melting process of n-octadecane was noticed. At this temperature, the temperature increase rate *R*_T_ of the PCM-18 macrocapsule package decreased from *R*_T_^0−3^ = 2.8 °C·min^−1^ to *R*_T_^3−7.5^ = 1.2 °C·min^−1^. The n-octadecane melting process continues for approximately 7.5 min, at which point the PCM-18 package reaches a temperature of 32.9 °C. After this time, the n-octadecane is completely melted, and the heating rate of the PCM-18 package increases to *R*_T_^7.5−10^ = 3.2 °C·min^−1^. In the experiment with exposure of macrocapsules to radiant heat, a large difference in the duration of the mixed state of PCM was observed between the two types of macrocapsules, similar to the experiment with exposure of macrocapsules to contact heat.

The PCM-18 macrocapsule package heated from an initial temperature of 19 °C and after approximately 3 min reached a temperature of 26.9 °C, at which the melting process of n-octadecane was noticed. At this temperature, the temperature increase rate *R*_T_ of the PCM-18 macrocapsule package decreased from *R*_T_^0−3^ = 2.8 °C·min^−1^ to *R*_T_^3−7.5^ = 1.2 °C·min^−1^. The n-octadecane melting process continues for approximately 7.5 min, at which point the PCM-18 package reaches a temperature of 32.9 °C. After this time, the n-octadecane is completely melted, and the heating rate of the PCM-18 package increases to *R*_T_^7.5−10^ =3.2 °C·min^−1^. In the experiment with exposure of macrocapsules to radiant heat, a large difference in the duration of the mixed state of PCM was observed between the two types of macrocapsules, similar to the experiment with exposure of macrocapsules to contact heat.

The radiant heat flux incident on the packets showed that the melting time of n-hexadecane in PCM-16 macrocapsules was approximately 3 min, while the melting time of n-octadecane in PCM-18 macrocapsules was 4.5 min. The observed 1.5-min difference in melting time may be due to the factors mentioned in the discussion of the results of the experiment with exposure of the packets to contact heat. In the experiment with exposure to radiant heat, the difference in melting time between the two PCMs is smaller, which may be related to the different heat flux values in the two experiments and the different heat transfer mechanisms.

## 4. Conclusions

The aim of this study was to evaluate macrocapsules containing phase change materials (n-hexadecane and n-octadecane) for potential applications in various areas. To determine the internal structure of the macrocapsules, statistical analysis of their microstructure was performed using high-resolution X-ray microtomography (micro-CT). Differential scanning calorimetry (DSC) was then used to determine the phase change temperatures, thermal stability, and phase transition enthalpies of both PCMs. Additionally, the kinetics of phase transition of these PCMs were analyzed in experiments simulating their potential application environments, i.e., by exposing them to contact heat and radiant heat. Based on the obtained results, it can be concluded that:

- Microstructure analysis of both macrocapsules showed that PCM-16 macrocapsules contain 11% less phase change material than PCM-18 macrocapsules (n-hexadecane constitutes 77% of the macrocapsule volume while n-octadecane constitutes 88% of the macrocapsule volume).
- DSC analysis involving heating and cooling cycles of macrocapsules showed that the tested PCMs exhibit good thermal cycling stability, which was confirmed by studies conducted by other researchers. PCM-18 exhibited a significantly wider phase transition range and a greater ability to store latent heat, indicated by its higher enthalpy by 30 J·g^−1^ than PCM-16.
- The analysis of phase transition processes in the experiment in which macrocapsules were exposed to contact heat showed that PCM-18 macrocapsules were characterized by a higher heat capacity, melting of which lasted more than three times longer than in the case of PCM-16 microcapsules.
- The analysis of phase transition processes in the experiment in which macrocapsules were exposed to radiant heat showed that PCM-18 macrocapsules were characterized by a higher heat capacity, the phase transition (melting) of which lasted more than 1.5 times longer than in the case of PCM-16 microcapsules.
- The temperature ranges of phase transitions of PCMs and their thermal cycling stability in both types of macrocapsules determined by DSC analysis and two experiments in which the macrocapsules were exposed to two types of heat flux show the potential application of tested PCMs in the thermal packaging industry, thermal insulation building materials, or thermal protective clothing. Modifying the above-mentioned products with these PCMs can improve their functional and utility properties and thus contribute to increasing people’s safety and comfort.

## Figures and Tables

**Table 1:** Physical properties of tested macrocapsules [24,25,26,27].

Macrocapsule	Raw Material	Specific Heat [kJ·kg^−1^·°C^−1^]	Melting Temperature [°C]	Heat of Fusion [kJ·kg^−1^]	Thermal Conductivity [W·m^−1^·°C ^−1^]	Diameter [mm]
PCM-16	C_16_H_34_ n-hexadecane (PCM) Polyurethane (shell)	1.8 (solid)/2.2 (liquid) 1.7	18.2 210	232	0.26 (solid)/0.14 (liquid) 0.19	3.8
PCM-18	C_18_H_38_ n-octadecane (PCM) Polyurethane (shell)	1.9 (solid)/2.2 (liquid) 1.7	28.2 210	243	0.31 (solid)/0.15 (liquid) 0.19	4.5

**Table 2:** Thermal properties of PCM-16 and PCM-18 macrocapsules determined using DSC method.

Macrocapsule	*T*_Mb_ °C	*T*_Me_ °C	∆*H*_M_ J·g^−1^	*T*_Mmax_ °C	*T*_Cb_ °C	*T*_Ce_ °C	∆*H*_C_ J·g^−1^	*T*_Cmax_ °C
PCM-16	15.3	22.7	150	18.9	14.7	6.2	−153	11.6
PCM-18	31.8	41.6	183	37.8	34.9	19.1	−187	30.2

## Data Availability

The data supporting the reported results are not stored in any publicly archived datasets. The readers can contact the corresponding author for any further clarification of the results obtained.
